# Liver Zonation – Revisiting Old Questions With New Technologies

**DOI:** 10.3389/fphys.2021.732929

**Published:** 2021-09-09

**Authors:** Rory P. Cunningham, Natalie Porat-Shliom

**Affiliations:** Thoracic and GI Malignancies Branch, Center for Cancer Research, National Cancer Institute, NIH, Bethesda, MD, United States

**Keywords:** liver, physiology, zonation, architecture, technologies

## Abstract

Despite the ever-increasing prevalence of non-alcoholic fatty liver disease (NAFLD), the etiology and pathogenesis remain poorly understood. This is due, in part, to the liver’s complex physiology and architecture. The liver maintains glucose and lipid homeostasis by coordinating numerous metabolic processes with great efficiency. This is made possible by the spatial compartmentalization of metabolic pathways a phenomenon known as liver zonation. Despite the importance of zonation to normal liver function, it is unresolved if and how perturbations to liver zonation can drive hepatic pathophysiology and NAFLD development. While hepatocyte heterogeneity has been identified over a century ago, its examination had been severely hindered due to technological limitations. Recent advances in single cell analysis and imaging technologies now permit further characterization of cells across the liver lobule. This review summarizes the advances in examining liver zonation and elucidating its regulatory role in liver physiology and pathology. Understanding the spatial organization of metabolism is vital to further our knowledge of liver disease and to provide targeted therapeutic avenues.

## Introduction

One of the central roles of the liver is maintaining energy homeostasis by preserving blood glucose levels. It does this by storing carbohydrates in the form of glycogen when nutrients are available, and then releasing it back into the blood stream during periods of fasting (Trefts et al., 2017). Facilitating this, the liver alternates between oxidizing fat or glucose for energy, depending on fuel availability. Additionally, the liver is one of the central hubs for protein synthesis – contributing to a wide array of circulating proteins such as albumin, and blood clotting factors. The liver also forms the first line of defense for drug and alcohol detoxification and is an important immunological organ. With these diverse functions in mind, the pertinent question remains – how can these varied and divergent processes coincide simultaneously in the liver?

**FIGURE 1:**
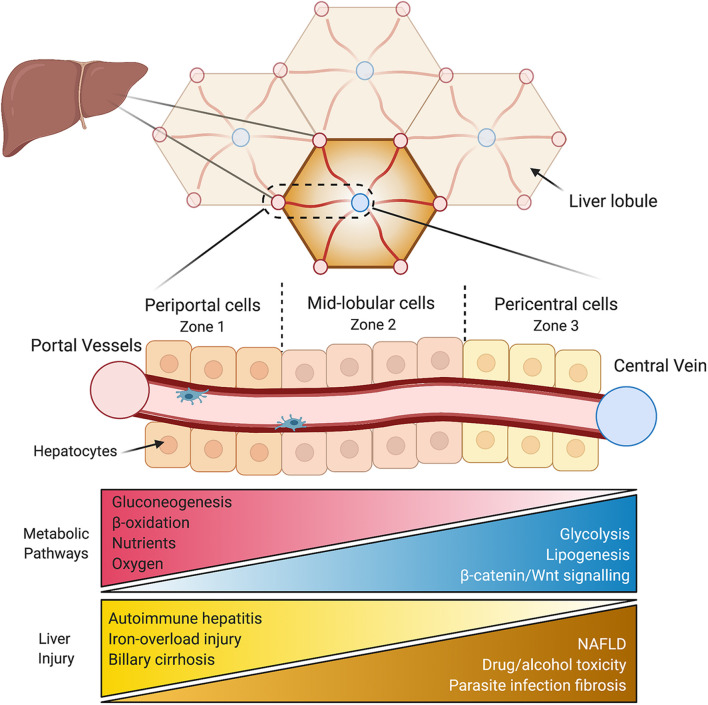
Liver anatomy and the hepatic lobule. The liver is composed of hexagonal units called lobules. Oxygen and nutrient rich blood flows directionally from the hepatic vessels (red) in the corners of the lobule toward a central vein in the middle (blue). Periportal hepatocytes (zone 1) are at the periphery of the lobule, followed by mid-lobular cells, and finally pericentral hepatocytes surround the central vein (zone 3). The variable microenvironment along the periportal-pericentral axis results in graded gene expression and the spatial separation of certain metabolic processes to periportal (red) and pericentral (blue) regions. Certain liver injuries are also zone-dependent, with some originating or confined to periportal (yellow) regions and some to pericentral regions of the lobule (brown). NAFLD, non-alcoholic fatty liver disease.

These multi-faceted, vital physiological roles are enabled by the liver’s complex architecture. The liver contains a vast array of cell types all interacting with each other, of which hepatocytes make up ∼60%. Spatially, hepatocytes are arranged into hexagonal units called lobules, made up of around 15 concentric layers of cells. Oxygen, hormone, and nutrient rich blood arrives through the hepatic artery and portal vein and enters at the periphery of the lobule, flowing toward a single central vein (Figure 1). Conversely, bile flows outward from the center of the lobule and drains in the peripheral portal bile duct. The regions surrounding the hepatic arteries and portal veins are known as periportal (zone 1), while those adjacent to the central vein are called the pericentral areas of the lobule (zone 3), with the cells in between these regions, referred to as mid-lobular hepatocytes (zone 2). As blood flows directionally toward the central vein, hepatocytes take up oxygen, nutrients, and metabolize hormones, actively shaping their microenvironment by creating a gradient along the periportal-pericentral axis. In turn, this gradient is one of the primary drivers of differential gene expression and the subsequent functional heterogeneity of cells within the lobule. As a result, the spatial metabolic compartmentalization allows opposing metabolic functions to operate simultaneously. For example, through non-uniform expression of the enzymes, glutamine synthesis is confined to only two layers of hepatocytes surrounding the central vein, whereas ureagenesis exclusively restricted to periportal areas (Gebhardt, 1992). Any ammonia escaping ureagenesis in periportal cells is consumed and converted to glutamine in pericentral cells, thereby maintaining low levels of ammonia. In addition to this binary (on/off) zonated pattern, other enzymes have a graded expression pattern across the lobule. For example, enzymes involved in lipid synthesis are expressed predominantly in the pericentral region and expression gradually diminishes toward the periphery of the lobule, while β-oxidation enzymes in periportal cells decrease in expression in a graded manner toward the central vein (Gebhardt, 1992). These two patterns of gene expression highlight the complexity and specificity of spatially separated functions in the lobule.

Examining liver zonation is vital for our understanding of liver disease, of which many develop non-uniformly across the lobule. One example is non-alcoholic fatty liver disease (NAFLD), a spectrum of liver disease, encompassing hepatic steatosis, steatohepatitis (NASH), cirrhosis, and its end stage of hepatocellular carcinoma (HCC), with disease progression often occurring in a zonated pattern. NAFLD and NASH/cirrhosis initially develop with hepatic steatosis and inflammation, respectively, in the pericentral cells and progresses outward (Chalasani et al., 2008; Yeh and Brunt, 2014). Similarly, drug and alcohol-induced hepatotoxicity (Gebhardt, 1992) as well as parasite infection-induced hepatic fibrosis (Loose et al., 1978) initiates in the pericentral area. Conversely, many liver injuries are restricted to or at least begin in periportal areas, such as autoimmune hepatitis (Sahebjam and Vierling, 2015), primary biliary cirrhosis (Lindor et al., 2009), and iron overload-induced injury (Mori et al., 2020). Moreover, patients with HCC display disrupted Wnt/β-catenin signaling (Tian et al., 2016), key regulators of liver zonation. Aside for non-uniform injury of hepatocytes, other hepatic cell types also display zonated pathology during disease progression to cirrhosis. A mouse model of fibrosis revealed that pericentral hepatic stellate cells differentiate to become the dominant pathogenic collagen-producing cells across the lobule (Dobie et al., 2019). Similarly, liver sinusoidal endothelial cells (LSECs) in pericentral regions are more susceptible to damage associated with liver cirrhosis compared to periportal LSECs, with increased capillarization and decreased regulation of endocytosis (Su et al., 2021). These zonated pathologies of various hepatic cell types reveal differential vulnerabilities which may conceal important underlying mechanisms. In other words, understanding why a disease develops in a certain region or zone may provide new details of its pathophysiology. Examination the non-uniform onset of liver disease, and liver zonation overall, is still in its infancy. Increasing our understanding of the spatial dependence of disease development and progression is crucial for creating targeted treatment approaches.

The concept of hepatic functional heterogeneity is gaining much attention, with several excellent reviews previously published on liver zonation (Jungermann and Katz, 1982; Gebhardt, 1992; Jungermann and Kietzmann, 1996; Gebhardt and Matz-Soja, 2014; Ben-Moshe and Itzkovitz, 2019). In this review, we highlight fundamental questions in liver biology and how addressing them provided insights into liver zonation. In parallel, we will explore how technological advances over the years and the bounds made in recent ones open new opportunity to investigate how zonation supports complex liver metabolism. While embracing technological advancements to further this exciting field, many of the questions raised a century ago are still not fully answered which will be underscored. Ultimately, an integrated understanding of the spatial and temporal regulation of liver function are urgently needed to deepen our knowledge of liver physiology and disease etiology that will enable more efficacious avenues of future treatment.

## Hepatocyte Cell Biology Across the Lobule

Cell biologists in the early 1900s were fueled purely by a passion for knowledge in the face of limited technology. Unperturbed by this lack of technological advancement, the study of hepatocyte cell biology in the lobule was initially based exclusively on electron microscopy in the early to mid 1900s. Noël (1923) divided the rat liver lobule into three zones of what he eloquently described as the zones of permanent activity (periportal), the intermediary zone (mid lobular), and the zone of permanent repose (pericentral). It was observed that hepatic mitochondria were larger and more spherical in the periportal zone and became elongated toward the central vein (Noël, 1923). Smith (1931) observed no lobular variation in rats with respect to mitochondria morphology, although pericentral cells tended to have less in number. Further, he described the deposition of glycogen after feeding starting from the periphery of the lobule toward the central vein, with its withdrawal during fasting in the opposite order. Kater (1933) used several animal models, including mice, rats, dogs, and birds, to describe the accumulation of glycogen and fat across the lobule, as well as alterations in mitochondria morphology. Deane (1944) showed larger mitochondria, Golgi, bile granules, glycogen, and less lipids in mouse periportal hepatocytes, while Novikoff (1959) described the heterogeneity of enzymes across the rat lobule. Indeed, Dean was the first to coin the term “zonation” when referring to hepatocyte heterogeneity as early as 1944 (Deane, 1944). These scientists were the pioneers of cellular biology at the time, and their work has spawned countless lines of research from these fascinating discoveries, not least being liver zonation. While astute observations for their time, they were limited to individual cell imaging and 2D rendering. This latter consideration may be of particular importance to mitochondrial morphology since a section through the middle of a slender mitochondrion would falsely give it a round and spherical shape. Given that many of the processes that are zonated in the lobule are mitochondria related, β-oxidation and oxidative phosphorylation to name two, examining the structural differences in this organelle and others would further the understanding of the spatial metabolic compartmentalization in the liver.

Recent improvements in imaging and mathematical modeling technology have overcome the limitation of 2D imaging of subcellular organelles. Using a combination of array tomography, transmission electron tomography, and 3D modeling technology, the ultrastructure and morphology of giant mitochondria were analyzed in livers from NAFLD patients (Shami et al., 2021). This analysis revealed the decreased surface area to volume ratio and disorganized cristae contribute to the mitochondrial dysfunction during NAFLD development, but unfortunately authors did not make comparisons between zones in the lobule. Another recent imaging innovation that can be utilized for the study of subcellular heterogeneity in the liver is focused ion beam scanning electron microscopy (FIB-SEM). This utilizes scanning electron microscopy to scan the surface of the sample, then the focused ion beam then “mills” the surface with nanometer precision. As a result, the increased resolution in the *Z*-axis allows examination of subcellular structure in great details. Recently, Parlakgül et al. (2020) resolved the three-dimensional organization of subcellular organelles using FIB-SEM imaging in conjunction with deep-learning-based image segmentation in the intact murine liver. Advancements made to FIB-SEM in recent years have increased imaging volume, allowing them to image large intact livers from both lean and obese mice to the scale of 15 full or partial hepatocytes. This analysis revealed that the hepatocyte endoplasmic reticulum undergoes significant structural re-organization during the transition from lean to NAFLD. The results underscore the dynamic nature of cellular organelles and their central role in metabolic adaptation and homeostasis. While authors used transmission electron microscopy to ensure that all hepatocytes were from the mid-lobular zone, using this sub-cellular resolution to examine periportal versus pericentral hepatocytes during NAFLD progression warrants further attention moving forward. These new imaging technologies allow for multi-scale imaging at high resolution from the subcellular to the lobule level that will surely yield new perspective to the century old questions.

## Hepatocyte Heterogeneity – From Discovery to Present

Early reports of hepatocyte heterogeneity were made in the first part of the 1900s and were purely observational and qualitative in nature (Noël, 1923; Smith, 1931; Kater, 1933; Deane, 1944). A seminal paper by Novikoff (1959) quantitatively described elevated gluconeogenic and mitochondrial respiratory enzymes in rat periportal cells via histochemistry, giving an early indication of functional heterogeneity across the lobule. As technological advances occurred, including the ability to separate the zones of the lobule, this proposed zonation of hepatocyte function was confirmed. Enzymatic assays and use of radiolabeled isolated sub-populations of hepatocytes isolated from rat livers revealed higher activity of gluconeogenesis and fatty acid oxidation in periportal hepatocytes, and elevated glycolysis and drug metabolism in pericentral cells (Nauck et al., 1981; Jungermann, 1983, 1988; Bengtsson et al., 1987; Guzmán et al., 1995). This functional heterogeneity and separation of periportal and pericentral cells are discussed in more detail in the next section.

These initial experiments were limited to investigating only a select few specific proteins and enzymatic activities at a time and did not shed light into the molecular basis underlying the heterogeneity. The ability to profile hepatocytes gene expression through mRNA analysis, enabled a higher throughput examination. In 2006, the gene expression profiles of isolated mouse periportal and pericentral cells examined via microarray analysis (Braeuning et al., 2006). These revealed for the first time, that 198 genes were differentially expressed in periportal and pericentral hepatocytes and supported previous findings of spatial separation of lipid and glucose utilization and synthesis. This study also revealed that transcriptional regulation is a major contributor to hepatocyte heterogeneity and further investigation is warranted to gain insight into the molecular makeup of different hepatocytes. The introduction of genome-wide RNA profiling known as bulk RNA sequencing (RNA-seq), enabled vastly higher throughputs – detecting thousands of gene transcripts. These datasets made it possible to aggregate cells into clusters based on their similarity in gene expression patterns. This advancement has paved the way for examination of hepatocyte heterogeneity and other cell types within the liver. Using bulk RNA-seq, human hepatocyte gene expression profiles have been created showing that periportal and pericentral zones are regulated in part, by gut derived toxins and xenobiotic metabolisms, respectively (McEnerney et al., 2017).

Single cell (sc) RNA-seq technologies now allow a window into hepatocytes differential gene expression at an even greater resolution. Halpern et al. (2017) performed scRNA-seq on dissociated hepatocytes from murine livers. Next, they utilized established zonated landmark genes to create a map, where hepatocytes can be retrospectively returned to their spatial position/layer within the lobule (Halpern et al., 2017). This allows for the high throughput of RNA sequencing while maintaining precise coordinates of gene expression distribution in the lobule. While increasing the spatial resolution of gene expression profiles, it is important to note that this is done retrospectively, *in silico*. Strikingly, they demonstrated that half of liver genes are non-uniformly expressed, namely zonated. Interestingly, the increased single cell resolution allowed the identification of genes that are highly expressed in mid-lobule layers. Two examples are *hamp* and *hamp2* that regulate hepatic iron levels, that would have otherwise been lost using binary periportal/pericentral classifications or investigating whole liver homogenate. Validation of the scRNA seq results were confirmed by smFISH to locate the spatial position of hepatocytes expressing the gene. In subsequent recent studies of human livers, scRNA-seq were performed on whole liver (Aizarani et al., 2019), malignant and non-malignant liver tumors (Massalha et al., 2020), and hepatic non-parenchymal cells (MacParland et al., 2018), revealing intricate zonated profiles of gene expression and the impact of cell-to-cell interactions has on gene expression. The use of scRNA seq provides the fundamental basis to understand gene expression patterns as the foundation underlying hepatocyte heterogeneity on which deeper understanding can be gained through the regulation at the protein level and interactions between different cells.

RNA-seq technologies allowed greater throughput than previous methods; however, the exact location/origin of the cell is lost during sample preparation and the dissociation process can introduce changes to gene expression (van den Brink et al., 2017; Saviano et al., 2020). To overcome these challenges, examining hepatic zonation in intact liver tissue would allow maximal preservation of the cellular metabolic state while maintaining spatial resolution. Filling this methodology gap, spatial transcriptomics expands upon traditional single cell or bulk sequencing, where cells lose their positional information once they are collected for cell-based RNA seq techniques. Spatial transcriptomics allows for the investigation of cells and tissues gene expression be examined while maintaining their spatial positioning. Particularly, *in situ* spatial transcriptomics has been developed to create a map of the cells’ location within the tissue prior to lysing (Rodriques et al., 2019; Vickovic et al., 2019). Here, tissue is placed on a slide with unique barcodes to define the cells’ position on the slide. After a standard hematoxylin and eosin-stained image, the tissue is lysed for scRNA-seq, and then the transcription profile of each cell is repositioned in space to its spatial barcoded position based on the original H&E image. This new technique has not yet been performed on liver sections, despite its great potential for investigation of liver zonation. This may be particularly useful for investigating heterogeneity in small sections of tissue, a common occurrence with human liver biopsies, where both transcriptomics and spatial positioning can be derived from a single tissue section without material loss.

Examining liver zonation using RNA-seq approaches allow high-throughput but are limited to transcriptional messaging. Expanding on their previous work of scRNA-seq, the Itzkovitz group developed spatial sorting of murine hepatocytes based on differential expression of cell surface markers, followed by proteomic analysis (Ben-Moshe et al., 2019). They created a comprehensive proteomic map and found a strong correlation between gene and protein expression. This indicates the prevailing regulation of hepatocyte heterogeneity occurs at the transcriptional level. However, several genes displayed conflicting expression between mRNA and protein – some genes were zonated at mRNA but not protein levels (*A1cf*, *Clmn*, and *Lsr*), while some were zonated at the protein but not the mRNA level (Rpb4, Idh3, and Mrpl43). Arguably, zonation at the protein level has a greater impact on hepatocyte function and dysfunction; therefore, examining mRNA only may cause important regulatory proteins to be missed. One of the challenges in high throughput evaluation of protein expression via Mass Spectrometry is sample size and sensitivity that may cause low abundance proteins to remained undiscovered. Beyond gene or protein expression, changes to post translational modifications likely come into play, adding an additional layer of regulation. Indeed, these are significantly harder to measure and would require further technological advancements. To fully understand the molecular basis of heterogeneity across the lobule, all dimensions of regulation must be considered and integrated. Moving forward, investigating how proteomics and post translation modifications shape the spatial compartmentalization of metabolic functions should help to fill any knowledge gaps left by examining transcriptional regulation only.

## Location, Location, Location – Spatial Division of Labor in the Lobule

The phrase “divide and conquer” succinctly describes the liver’s unique ability to spatially separate opposing functions for increased metabolic efficiency. Examining this division of labor across the lobule was made possible through extensive technological advances. Initial observations were made by immunohistochemical studies, where the expression of certain enzymes served as a proxy for function. Specifically, the pioneering work by Jungermann demonstrated the spatial division of carbohydrate metabolism across the rat liver lobule, with gluconeogenic enzymes predominantly localized in periportal regions and glycolytic enzymes located in pericentral regions (Katz et al., 1977a,b). However, the mere expression of an enzyme does not necessarily translate into activity. This was addressed by elegant radiolabeling and flux rate studies confirming the dominance of gluconeogenesis and glycolysis in rat periportal and pericentral hepatocytes, respectively (Katz and Jungermann, 1976; Jungermann et al., 1982). The ability to separate hepatocytes into their respective zones was paramount in assessing differences in hepatocyte function and to move away from purely observational distribution of enzymes. One of the earliest attempts was made by Shank et al. (1959) using a dissecting microscope. Guided by the portal tracks as a landmark, they dissected periportal, midlobular, and pericentral hepatocytes from frozen sections of rat liver followed by biochemical analysis (Shank et al., 1959). The measured higher activity of glucose-6-phosphate dehydrogenase and lactate dehydrogenase periportal areas, while isocitric and glutamic dehydrogenase activity were elevated in pericentral cells. This method was the foundation on which laser capture microdissection (LCM) was developed, which utilizes a laser beam instead of a dissecting microscope for increased precision to isolated and classify certain zones of the liver (Saito et al., 2013; McEnerney et al., 2017). Using LCM to separate zones and pathway analysis with RNA sequencing data, McEnerney et al. (2017) demonstrated in human liver lobules that immune response pathways were localized to periportal regions while xenobiotic metabolism is predominantly in pericentral cells. One caveat of this approach is the inability to distinguish between hepatocytes and non-parenchymal cells. As such, Kupffer cells, stellate cells, and liver sinusoidal endothelial cell (LSECs), all of which display non-uniform distribution (Halpern et al., 2018; Gola et al., 2021), may confound the results.

Selective zonal damage has also been used to isolate hepatocytes form different zones for functional analysis using ortho (perfusion via portal vein) and retrograde (perfusion via central vein) delivery of harmful agents. Perfusion of the detergent digitonin via the portal vein, will result in selective damage to periportal cells while retrograde perfusion of collagenase will dissociate and release pericentral cells that can be functionally analyzed *in vitro*. Similarly, digitonin perfusion via the vena cava maintains viable periportal cells. First described in rats in 1985 (Lindros and Penttilä, 1985; Quistorff, 1985), the digitonin-collagenase perfusion technique has been used to evaluate enzymatic activity in the isolated cells that demonstrated that drug metabolism is restricted to pericentral cells (Bengtsson et al., 1987). Further, radiolabeled fatty acid oxidation experiments in rats revealed profound zone flexibility in fatty acid oxidation in response to a variety of conditions (Guzmán et al., 1995). Specifically, fatty acid oxidation was higher in periportal hepatocytes compared to pericentral cells under both fed and fasted conditions, confirming previous reports of higher β-oxidation in periportal hepatocytes. Additionally, pericentral hepatocytes relied on non-mitochondrial fatty acid oxidation, as peroxisomal fatty acid oxidation was elevated in these cells across all conditions. Unfortunately, digitonin-collagenase perfusion relies on damaging one cell population while recovering another, forcing the comparison between zones to be made between separate animals and increasing variability. Further, isolation may take several hours, resulting in one population of cells to be *ex vivo* several hours longer. These limitations were demonstrated when periportal and pericentral cells were isolated from two different lobes from the same rat liver at the same time (Tordjmann et al., 1997). Here, authors showed that sensitivity to angiotensin II was up to 80% higher in pericentral cells versus periportal, whereas no differences between zones had been recorded previously. Further, albumin mRNA was only 35% higher in periportal cells, as opposed to previously larger reported differences from cells isolated from separate animals. These data reveal that periportal and pericentral cells isolated from two different livers increase further variability between the zones and may significantly mask or alter zonated markers.

Separating periportal and pericentral cells via fluorescent-activated cell sorting (FACS) has also been used for decades (Gumucio et al., 1981; Thalhammer et al., 1989). In this form of flow cytometry, proximal and distal zones of the lobule are stained via ortho and retrograde perfusion before this homogenate of isolated hepatocytes are separated by FACS based on their fluorescent zone-specific labeling. Using this method, Thalhammer et al. (1989) demonstrated that succinate dehydrogenase and β-glucuronidase activity (related to oxidative phosphorylation and carbohydrate breakdown, respectively) were elevated in periportal cells compared to pericentral cells in rats. While this method allowed comparisons of hepatocyte subpopulations in the same liver, it was still constrained to the accuracy and the length of time it required for forward and backward perfusions. Greatly enhancing the separation efficiency of hepatocyte sub-types via FACS has been the identification of bona fide periportal and pericentral protein markers. Using perfused murine livers to dissociate cells, isolated hepatocytes stained with pericentral (CD73 or glutamine synthetase) and periportal (E-cadherin) were categorized into periportal or pericentral zones based on their positive cell populations for certain zonated markers (Halpern et al., 2017; Ben-Moshe et al., 2019; Berndt et al., 2021). The Itzkovitz group further stratified these hepatocytes into eight layers by defining “gates” based on their combined fluorescence of their pericentral or periportal markers (CD73 and e-cadherin) (Ben-Moshe et al., 2019). These gates were then validated against their spatially resolved scRNA-seq map (Halpern et al., 2017). Notably, this method increases the spatial resolution of protein expression profile by stratifying hepatocytes across different layers of the lobule instead of the binary classification of periportal and pericentral cells. Combining FACS, proteomics and pathway analysis, the Itzkovitz group colleagues demonstrated in mice that energetically demanding tasks such as plasma protein production and oxidative phosphorylation pathways were elevated in periportal regions, while xenobiotic and glutathione metabolism were pericentrally located (Ben-Moshe et al., 2019). Despite this advancement, *in vivo* functional relevance can be difficult to infer from gene and protein expression only. Addressing this, a shotgun proteomic approach on zone-specific hepatocytes isolated via immunostaining and subsequent FACS was used to explore the proteome map of the mouse liver lobule (Berndt et al., 2021). Relative protein abundance of enzymes and membrane transporters in pericentral and periportal hepatocytes were quantified to infer the functional significance using biochemistry-based kinetic model of hepatocyte metabolism (Berndt et al., 2018). This model demonstrated that proteins and enzymes related to glycolysis were elevated in pericentral cells, whereas periportal cells had a higher gluconeogenic capacity. Periportal hepatocytes also had elevated respiratory chain complexes, indicating a higher capacity for ATP generation, confirmed by the model demonstrating higher ATP/ADP rations in periportal versus pericentral cells.

**FIGURE 2:**
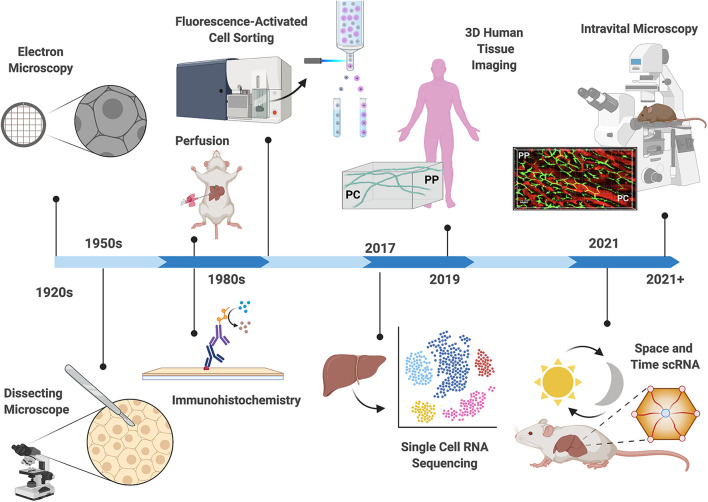
Technological advances in the study of liver zonation. Over the past century, technological advances have driven discoveries related to hepatocyte organization and function. Early observations describing structural heterogeneity were confined to microscopy techniques (Noël, 1923; Kater, 1933; Shank et al., 1959). The development of hepatocyte separation approaches (1950–1980), including perfusion techniques, fluorescent activated cell sorting (FACS), and immunohistochemistry allowed biochemical classification of hepatocyte activities (Baron et al., 1981; Gumucio et al., 1981; Lindros and Penttilä, 1985). Deep insight into the differential gene expression patterns in the hepatic lobule was accomplished in 2017, where single cell RNA sequencing revealed that fifty percent of liver genes are non-uniformly distributed (Halpern et al., 2017). The first three-dimensional (3D) multiphoton microscopy of the human liver lobule reveled disrupted bile canalicular network in nonalcoholic fatty liver disease patients in 2019 (Segovia-Miranda et al., 2019). Recently (2021), changes in gene expression patterns across the lobule were examined in different times of the day revealing yet another layer of complexity in the regulation of compartmentalization (Droin et al., 2021). Advances in mouse genetics and light microscopy now allow the use of Intravital microscopy for examination of the hepatic lobule in real time. Depicted is a projection of 3D volume of the murine lobule acquired with intravital confocal microscopy (PP, periportal; PC, pericentral; bile canaliculi in green, sinusoids in red).

Overall, the ability to examine the spatial division of labor across the lobule had made significant strides in the last century (Figure 2). This is due, in part, to improved ability to separate periportal and pericentral cells from the same animal at the same time. Further, proteomic analysis with increased spatial resolution now allows high throughput investigation of enzyme expression which is relevant to the functional capacities in different zones. Moving forward, *in vivo* models to measure metabolic functions in intact livers, and how they change in response to perturbation will shed light on the spatial and temporal regulation of liver function.

## What Regulates Liver Zonation?

Hepatocytes are organized in an assembly-line-like structure giving rise to speculations regarding the role liver anatomy fulfills in zonation. Specifically, periportal and pericentral regions of the lobule are exposed to different environments: blood entering at the periphery of the lobule is rich in oxygen and nutrients, whereas pericentral cells are exposed to more hypoxic and nutrient sparce conditions (Rappaport et al., 1954). Deane (1944) intuitively speculated that many of the changes in glycogen storage and organelle morphology she observed, were due to the higher levels of oxygen in periportal regions of mice. In depth analysis of the liver anatomy by Rappaport in the 1950’s elegantly described how the hepatic vascular framework plays a major role in dictating both the structure and function of the mammalian liver (Rappaport, 1958, 1973). This vascular architecture is regulated by a multitude of factor such as hydrostatic pressure and the microvasculature smooth muscle contractility which respond to nervous stimuli, hormones and metabolites, among others. Indeed, the regulation of carbohydrate metabolism across the lobule by oxygen and the glucagon/insulin ratio was elegantly demonstrated by Jungermann both *in vitro* and perfused rat livers (Nauck et al., 1981; Wölfle et al., 1983; Wölfle and Jungermann, 1985; Jungermann and Kietzmann, 1997, 2000), supporting the role of nutrient and metabolic hormone gradients in the regulation of hepatocyte gene expression and functions.

The introduction of transgenic mouse lines has greatly increased the ability to examine factors regulating liver zonation through tissue-specific loss- and gain-of function studies. Conditional knockout of β-catenin and its negative regulator adenomatous polyposis coli (APC), demonstrated the essential role of β-catenin/Wnt signaling in liver zonation (Benhamouche et al., 2006; Sekine et al., 2006). Interestingly, β-catenin and its negative regulator APC were shown to be expressed in pericentral and periportal regions, respectively (Benhamouche et al., 2006). Knockout of APC, which activates β-catenin, imposed a pericentral like phenotype across the lobule as identified by immunohistochemistry and microarray analysis of zonated pathways. Conversely, β-catenin KO mice displayed elevated periportal features in the lobule and loss of pericentral ones including disrupted ammonia and xenobiotic metabolism. Remarkably, blocking upstream Wnt signaling via *Lpr5* knockout mice resulted in the same disruption of hepatocyte heterogeneity as β-catenin knockout mice (Yang et al., 2014), indicating that β-catenin is regulated by Wnt signaling. Instead of hepatocytes, Kupffer cells and endothelial cells surrounding the central vein were found to be the source of Wnt ligand secretion, highlighting the complex intracellular crosstalk required for maintaining liver zonation (Preziosi et al., 2018). Counterbalancing pericentral zonation phenotype of β-catenin/Wnt signaling, bloodborne molecule/s activate Ras signaling that in turn promote the expression of periportal genes while suppressing pericentral associated genes (Braeuning et al., 2007). Another antagonist to the β-catenin/Wnt signaling pathway is glucagon, which exhibits a declining concentration gradient like that of oxygen along the periportal to pericentral axis. Glucagon deficient mice exhibit perturbed zonation profiles with dampened periportal gene expression and extended glutamine synthase expression, indicating a shift toward a more pericentral like phenotype (Cheng et al., 2018). Moreover, reinfusion of glucagon in deficient mice restored liver zonation profiles. This push and pull relationship between glucagon/Ras and β-catenin/Wnt signaling create a fine balancing act that shape metabolic compartmentalization across the lobule. The fact that glucagon is secreted in response to fasting underlines the livers’ ability to quickly alter its zonation profile in order to adapt to substrate availability.

Oxygen gradient across the lobule drive transcriptional responses that are regulated by hypoxia-induced transcription factors (HIFs). Those are predominantly active in pericentral regions where oxygen concentrations are markedly lower (Kietzmann et al., 2001). HIFs provide another layer of regulation for liver zonation and patterned gene expression profiles due to their crosstalk with other important factors governing heterogeneity (Kietzmann, 2019). For example, ablation of the *hif-1a* gene in stem cells reduced β-catenin/Wnt gene expression in hypoxic conditions (Mazumdar et al., 2010), and β-catenin’s negative regulator, APC, directly represses HIF-1α (Newton et al., 2010). This interplay between factors regulating liver zonation underlines the complexity of this process but also highlight critical regulatory junctions that warrants further investigation in liver pathophysiology.

These earlier discoveries of factors that regulate liver zonation were examined using new technologies. Single cell RNA-seq revealed Wnt signaling, and low oxygen are drivers of pericentral zonation profiles, while Ras signaling being a key inducer of periportal zonation, and pituitary hormones repress pericentral genes (Halpern et al., 2017). Notably, two thirds of zonated genes were not targets of Wnt, hypoxia, Ras signaling, or pituitary hormones, indicating that a combination of these factors or other unknown factors are at play highlighting how much more there is to be discovered. For example, exercise and physical activity is known to improve NAFLD outcomes and increase hepatic metabolic efficiency (Rector et al., 2008; Stevanović et al., 2020; Thyfault and Rector, 2020). Whether the effect of exercise is ubiquitous across the lobule or whether it induces a zone-specific phenotype are not known.

Indeed, new technologies now allow the discovery of new factors that shape liver zonation. Recently, the microbiome was shown to help establish a periportal dominant immune zonation in the mammalian lobule (Gola et al., 2021). Here, LSECs sensed gut derived bacteria which in turn triggered a signaling cascade and chemokine secretion which ultimately orchestrated immune cell localization to periportal regions. This novel discovery was made possible via a combination of advanced techniques such as multiplex imaging of human tissue, transgenic mouse lines, and transcriptomics. This finding opens the door for further investigation as to additional factors of liver zonation that may not have been previously considered and exemplifies the complex interplay that regulates hepatic heterogeneity.

## Do Non-Parenchymal Cells also Display Heterogeneity Within the Lobule?

Like hepatocytes, non-parenchymal cells are also exposed to distinct microenvironments created by gradient of factors across the lobule. It is therefore likely that these gradients shape their gene expression, morphology, and function. One example is zonation of LSECs morphology which shows larger and more porous cells in the pericentral zone of rats, potentially facilitating filtration of toxins (Blouin et al., 1977; Gebhardt, 1992). A second example is related to enrichment of immune cells in periportal regions and the important central role this fulfills in proper immunological response. Indeed, initial histological and microscopy reports in rodents observed the liver’s resident macrophage, Kupffer cells, to be predominantly located in periportal regions of the rat lobule (Bouwens et al., 1986; Lough et al., 1987). These qualitative reports were confirmed and expanded upon by the use of scRNA-seq and immunohistochemistry, demonstrating that Kupffer cell gene expression was upregulated in periportal areas in mice (Halpern et al., 2017) and humans (MacParland et al., 2018). Advancements in imaging technology has allowed further characterization of hepatic immune cell heterogeneity – two-photon intravital microscopy revealed natural killer T cells to be concentrated around periportal areas (Geissmann et al., 2005). In an elegant study by Gola et al. (2021), clearing-enhanced 3D imaging showed Kupffer cells and natural killer T cells enriched in periportal regions of mice and human lobules. Further, they demonstrated that this immune cell zonation was driven by the exposure of gut derived bacteria sensed by LSECs and the formation of chemokine gradients across the lobule, rather than classic Wnt signaling. When immune cell positioning and zonation was disrupted, authors observed increased bacterial spread toward pericentral regions and inflammatory damage, highlighting the importance of the non-uniform distribution of resident immune cells across the lobule. Collectively, these data indicate that hepatic immune cell heterogeneity is centralized in periportal zones of the lobule to prevent bacteria from reaching pericentral regions and entering the blood stream.

One of the difficulties in mapping any potential zonation profile of non-parenchymal cells in the liver is due in part, to the high abundance of hepatocytes masking any heterogeneity of less abundant cell types. LSECs are one such cell type, and often lack sufficient material for RNA-seq techniques or well-established landmark zonation genes. Gola et al. (2021) mapped the location of LSECs across the lobule using only the expression of CD117, with higher gradient of expression from periportal to pericentral regions. However, using hepatocyte zonation markers to help guide the zonation of LSECs would give a more comprehensive zonation map. Applying this concept, Halpern et al. (2018) developed paired-cell RNA sequencing (pcRNA-seq), where the zonation profile of LSECs is based on the profile of the hepatocyte that is attached to it from the previously established hepatocyte scRNA-seq data (Halpern et al., 2017). This approach revealed that approximately 35% of LSECs genes in mice were zonated, a fact that would be otherwise masked by the significantly more abundant hepatocyte gene expression. Interestingly, the authors demonstrated that pericentral LSECs expressed both Wnt ligands and the Wnt antagonist Dkk3, indicating a fine balancing act in the pericentral region of both positive and negative Wnt regulators. These data suggest that LSECs, like hepatocytes, are highly zonated, and that pcRNA-seq is a viable method for examining low abundant cells populations across tissues. Supporting these findings, recent pathway analysis on RNA-seq data and immunohistochemistry from human liver samples have identified distinct sub-population of LSECs across the liver lobule with functional heterogeneity depending on their location (Strauss et al., 2017; MacParland et al., 2018). Strauss et al. (2017) referred to periportal LSECs as type 1 and LSECs in midlobular and pericentral zones as type 2. In addition to having a different molecular phenotype, type 1 LSECs had a wider luminal space than type 2 LSECs to facilitate a reduction in blood flow velocity into the lobule. Non-parenchymal cells also display spatially dependent pathologies during progression to cirrhosis. Single cell analysis revealed that in a mouse model of liver cirrhosis, LSECs in pericentral areas are more susceptible to damage than periportal LSECs, and displayed increased capillarization and impaired regulation of endocytosis (Su et al., 2021). Further, pericentral hepatic stellate cells were identified as key collagen producing cells during liver fibrosis in mice (Dobie et al., 2019). This spatial zonation of hepatic stellate cells during cirrhosis development was due to only pericentral stellate cells differentiating into pathogenic collagen producing cells. Mapping out the spatial heterogeneity of all hepatic cell types is important to understand the full picture of metabolic compartmentalization in the liver, and whether disruption of non-parenchymal cell zonation is a significant contributor to disease development and progression.

## How Does Liver Zonation Change Over Time?

It is well established that hepatocyte function is altered by their position within the lobule, but is it also dependent on time of day? One of the earliest observations supporting this idea was made by Deane (1944). She excised mouse livers every 3 h for a 24 h period (Deane, 1944) and describes changes to mitochondrial morphologies in the periportal region in different times of the day. Consistently, other cellular components show zonal distribution that changes during the day. For example, bile granules were more numerous in periportal regions and peaked during the fasting hours. In contrast, lipid droplets are concentrated in pericentral cells but become more abundant and widely spread during fasting. Glycogen was deposited initially in the periportal areas in the hours post feeding, and then along the lobule toward the center (Uchiyama, 1990). Subsequently, extended fasting led to the depletion of glycogen storages from pericentral cells first. While these studies described important observations, they were limited by the temporal resolution (∼3 h intervals) and confined to qualitative data. Assessing gene and protein expression as well as organelle composition is vital to understanding how temporal parameters such as feeding/fasting cycles and circadian rhythms regulate liver zonation.

In recent years, circadian rhythm has been recognized as a robust driver of hepatic function and gene expression (Rey et al., 2011; Atger et al., 2015). However, the interaction between spatial and temporal aspects in liver physiology and pathophysiology is relatively unknown. Is liver zonation dependent on time of day? To answer these questions and expand on previous studies that were limited to qualitative analysis, the Itzkovitz group added another dimension to their spatial analysis of liver zonation – time. In collaboration with chronobiologist Felix Naef, scRNA-seq was performed on mouse hepatocytes across four different time points in a 24-h period with a 12/12 light/dark cycle (Droin et al., 2021). The authors found 30% of hepatocyte genes were regulated by space only, while some (20%) were affected only by time of day. For example, the core clock genes (*bmal1*, *dbp*) were unsurprisingly strongly regulated by temporal rhythms and expressed ubiquitously across the different zones, visually confirmed using smFISH. Interestingly, 7% of all genes displayed effects of both space and time; for example, the fatty acid synthesis and mitophagy genes *elovl3* and *bnip3*, respectively, both had higher expression in pericentral cells, whereas *elovl3* peaked in expression during the fed period and *bnip3* peaked during the fasting cycle. This dual regulation of expression in both time and space allows the liver to appropriately respond to metabolic demands and substrate availability during the diurnal feeding and fasting cycles. Further, key regulators of liver zonation – Wnt and hypoxia signaling, also displayed dual regulation, with targets of both peaking in expression in pericentral cells at the end of the fasting period. This indicates that temporal rhythm may also regulate hepatocyte heterogeneity, and that the metabolic switch between the feeding and fasting cycle may be an important consideration when examining liver zonation. While 7% of genes displayed effects of both space and time, only a minute fraction displayed interactions between these two variables. In other words, a small subset of zonated genes (either high pericentral or periportal expression) flipped their location depending on the time of day, or genes with strong temporal rhythms (high expression during the day, low at night) were reversed in different zones of the lobule. Whether these interactions between space and time gene regulation in the lobule are exacerbated in the setting of liver disease is unknown but warrants further investigation.

The advances of high throughput technology as well as the deeper understanding of the factors regulating temporal and diurnal rhythms have shown the complexity of the interactions between spatial and temporal regulation of liver zonation. While these studies demonstrate that liver zonation changes during the day, methods that allow high temporal resolution are lacking. Advances in light microscopy, such as intravital microscopy (IVM) has made it possible to examine cells in live tissue to provide spatial and temporal accuracy for physiological events *in vivo*. Recent advances in methodology to reduce motion artifact such as respiration and heartbeat have enabled examination of live tissue phenomena that would otherwise confined to *in vitro* (Weigert et al., 2013). Taking advantage of these improvements in imaging methods, fluorescence probes, and mouse genetics, Porat-Shliom et al. (2016) used IVM to demonstrate *in vivo* hepatic glucose uptake (Stefkovich et al., 2021), and how liver kinase B1 (LKB1) is a vital regulator of hepatocyte tight junctions. Mice lacking LKB1 had impaired hepatocyte transport kinetics and increased cellular permeability resulting from junctional disruption, all of which was visualized in real time using IVM. Moving forward, methods that can track *in vivo* processes across time such as IVM, will be vital for encompassing both spatial and temporal regulation of liver zonation.

## The Impact of Liver Zonation on Detoxification

Drug detoxification and metabolism, also known as xenobiotic metabolism, is one of the liver’s most vital functions for removing foreign chemicals (i.e., drugs, alcohol, and toxins). This has been one obstacle in developing drugs for treating liver disease, including NAFLD. Therefore, it is critical to understand hepatocytes that specialize in drug metabolism with the long-term goal to improve drug treatment efficiency. Initially described in the late 1970s and early 80s, hepatic detoxification was shown to be pericentrally localized via spectrophotometric and immunohistochemical analysis; higher expression of xenobiotic metabolizing enzymes, particularly cytochrome P-450, were in pericentral regions compared to periportal or mid-lobular in rat and human liver (Gooding et al., 1978; Baron et al., 1981; Hall et al., 1989; Bühler et al., 1992). Additionally, flow cytometry has been used to determine rat hepatocyte subpopulations based on the ability of cytochrome P-450 to metabolize ethoxy fluorescein ethyl ester to fluorescein (Miller, 1983; Black et al., 1993). Selective zonal damage has been particularly useful for examination of detoxification zonation, as acetaminophen overdose induce necrosis of pericentral regions only (Anundi et al., 1993; Ben-Shachar et al., 2012). In periportal and pericentral cells isolated via digitonin perfusion, pericentral cells exhibited higher cytochrome P-450 levels in response to acetaminophen, as well as increased vulnerability to drug induced injury when isolated from ethanol pre-treated rats (Anundi et al., 1993). Further, pericentral regions have higher markers of tissue damage after carbon tetrachloride-induced liver injury in mice (Abdel-Bakky et al., 2015). More recently, the use of scRNA-seq and smFISH confirmed these earlier reports, demonstrating that genes related to detoxification and drug metabolisms had elevated expression in pericentral regions in mice (Halpern et al., 2017). Similarly, pathway analysis of human hepatocyte gene expression cross referenced with established mouse zonation markers revealed an increase in detoxification related genes in pericentral zones suggesting that this spatial positioning is evolutionary conserved (MacParland et al., 2018).

To circumvent the challenges of using *in vivo* mouse models, mathematical and virtual models have been attempted to examine the heterogeneity of drug-induced liver injury. Robust pericentral accumulation of the N-acetyl-p-benzoquinone imine (NAPQI), a toxic byproduct of acetaminophen metabolism, explained the early pericentral necrosis during drug induced liver injury using virtual mice (Smith et al., 2016). A more recent mathematical model of acetaminophen induced liver injury suggested intrahepatocyte communication contributed to this explanation of pericentral dominant necrosis during drug induced injury (Kennedy et al., 2019). This intrahepatic communication suggested exosomes, gap junctions, and connexin hemichannels play an essential role in the toxic effect of chemicals, including facilitating or counteracting cell death processes.

Mathematical modeling has enabled the integration of multiple factors to identify testable hypotheses. However, the spatial and temporal complexity of xenobiotic metabolism make *in vivo* studies the gold standard. Tavakoli et al. (2019) used IVM to track the transport kinetics of fluorescein, a surrogate drug molecule; enabling the examination of live hepatic drug metabolism in the different zones of the rat liver and its interaction with hepatocytes, sinusoids, and bile canaliculi. The combination of IVM and mathematical modeling allowed the authors to accurately track and infer the kinetics of fluorescein across the different compartments in the liver. This novel technology has allowed for the first time, *in vivo* tracking of drug metabolism across the liver lobule – an enormous leap from the methods of 50 years ago. With hepatotoxicity the primary reason for failed phase I drug development (Larrey, 2002), the use of IVM for understanding how liver zonation affects the pharmokinetics of new therapeutics is an important and timely advancement. Further, using IVM avoids the issue of other cell types becoming damaged and compensatory changes to other zones of the liver during selective zonal damage techniques. Further investigation of hepatocytes that specialize in drug metabolism will greatly enhance our ability to design more effective and accurate pharmacological treatments for liver disease.

## Is Liver Regeneration Zone Specific?

The liver has the remarkable capacity of self-regeneration, however, the source of the new cells in hepatic homeostasis and regeneration is one of the unresolved mysteries in liver biology. Initial models addressing this question were of liver streaming – where new hepatocytes were generated in the periportal regions and slowly migrated toward the central vein (Zajicek et al., 1985; Lee et al., 1998). This concept has been controversial, with evidence suggesting that proliferating hepatocytes were distributed evenly across the lobule (Bralet et al., 1994). The introduction of Cre-loxP technology which allows gene editing, has greatly enhanced the ability to examine the process of regeneration. Transgenic mouse lines tracking hepatic *Lgr5*, a stem cell marker, revealed that hepatocytes around the central vein give rise to their own lineage and are self-sustaining during liver injury (Ang et al., 2019). *Lgr5* positive hepatocytes overlapped with hepatocytes expressing glutamine synthetase (pericentral marker), but not e-cadherin (periportal marker). In addition, the *Lgr5* positive cells were also found to be the source for HCC development. Supporting this, pericentral hepatocytes in mice have increased expression of liver progenitor markers and can support and replace most hepatocytes in the lobule during homeostatic renewal (Wang et al., 2015). In direct discordance with this, lineage tracing indicated that liver regeneration after partial hepatectomy in mice is carried out uniformly throughout the liver, as opposed to being driven by those in pericentral regions (Sun et al., 2020). Conversely still, periportal hepatocytes were found to drive regeneration and proliferation following liver injury in chronic HCC murine models (Font-Burgada et al., 2015). These discrepancies could indicate that distinct population of hepatocyte are responsible for renewal in response to different forms of injury.

The controversy surrounding hepatic regeneration may have been born out of methodological shortcomings. These studies typically rely on tracing of a single marker gene to trace the lineage of the cells resulting in a partial or skewed view of the process. Recently, new methods have enabled the unbiased tracing of multiple hepatocyte population with high temporal and spatial accuracy during regeneration. Incorporating dual recombinase genetic-mediated murine models and hepatocyte-specific promotors, He et al. (2021) were able to non-invasively monitor long-term hepatocyte proliferation in live mice by constitutively activating a recombinase that permanently records hepatocyte transcription. This genetic proliferation lineage tracing method (Protrace) along with light-sheet microscopy, identified midzonal hepatocytes to be the major contributors of proliferation during liver homeostasis, without having to rely on individual lineage tracing genes. Furthering this comprehensive examination of regeneration heterogeneity in the liver, 11 conditional Cre knock-in murine strains were generated to enable inducible fluorescent labeling of hepatocytes across zones of the lobule to compare the regenerative capacity (Wei et al., 2021). Like He et al. (2021), authors demonstrated that midlobular hepatocytes were the main source of hepatocyte regeneration during liver homeostasis. Single-cell and bulk RNA sequencing, as well as CRISPER knockout models were used to identify genes and pathways regulating this increased proliferative capacity of mid lobular hepatocytes. These methods identified insulin-like growth factor binding protein 2, mammalian target of rapamycin, and cyclin d1 as the major regulators of mid lobular regeneration. Moreover, it was demonstrated that regeneration in response to chronic biliary (periportal) and centrilobular injury is dependent on the damaged area; damage to periportal areas increases regeneration in mid lobular and pericentral cells, while damage to the pericentral area increases regeneration in periportal and midlobular areas.

These two groundbreaking studies challenge the notion that midlobular cells are merely a transition zone between periportal and pericentral hepatocytes and identify them as the main contributor to liver regeneration. It was speculated that this may be due in part, to their spatial protection from toxic injuries at either end of the lobule and are appropriately positioned to initiate regeneration to replenish both periportal and pericentral hepatocytes. Indeed, identifying the mechanisms underlying the regenerative capacity of mid-lobular cells is vital for our ability to restore function following liver injury and disease.

## Examining the Bile Canalicular Network Across the Liver Lobule

Bile acids are produced and secreted from the liver to emulsify dietary lipids and act as signaling molecules, before being recycled and reabsorbed by hepatocytes. They flow directionally from pericentral toward periportal which is in the opposite direction of blood flow. The bile canalicular network also display structural and functional heterogeneity along the liver lobule. Specifically, variations in canalicular size and branching were observed via electron microscopy (Layden and Boyer, 1978; Baumgartner et al., 1987). Functionally, uneven bile acid uptake and excretion across the lobule was observed by infusion of bile-acid analogs and autoradiography (Jones et al., 1980; Groothuis et al., 1982). In a series of experiments by Baumgartner et al. (1986, 1987), retrograde perfusions in rats showed labeled taurocholate to be excreted slower than during orthograde, indicating greater uptake in periportal hepatocytes. Experiments of selective damage to pericentral hepatocytes in rats confirmed that pericentral hepatocytes contributed only a small fraction of uptake, with the vast majority occurring in periportal and midlobular cells (Gumucio et al., 1979), and that the pericentral region is the prime area for bile acid synthesis (Dionne et al., 1990). While these studies demonstrated initial structural and functional canalicular heterogeneity, examining dynamic properties of the biliary fluid network were limited by technology at the time.

Significant strides in imaging, particularly multiphoton IVM, have allowed for in depth analysis of the biliary network. Meyer *at al*. performed IVM of the mouse biliary network and mathematical modeling to demonstrate that bile flow increases in speed from the central vein to the bile ducts in the periportal regions, while biliary pressure decreases in the same direction (Meyer et al., 2017). These data indicate that both bile canalicular contractility and osmotic pressure display important heterogeneity across the liver lobule to maintain bile flow dynamics. Furthering the understanding of bile canaliculi structure in human NAFLD patients, multiphoton microscopy and 3D tissue reconstruction was used to identify the pathologic adaptation of hepatic architecture and function across the spectrum of NAFLD development (Segovia-Miranda et al., 2019). Multiphoton microscopy has advantages over other forms of light microscopy modalities due to its deep tissue penetration and limited photodamage, making it an ideal method for imaging the complex, three-dimensional organization of intact tissue. Here, human liver samples were obtained from patients with varying degrees of NAFLD – healthy control, healthy obese, steatosis, and NASH. To determine different zone of the liver, the lobule was computationally divided into 10 equidistance regions along the pericentral to periportal region. 3D spatially resolved analysis revealed that lipid droplets were typically pericentrally located but expanded to periportal regions upon NASH progression. Further, the bile canaliculi network presented with disrupted integrity with NAFLD onset, an interesting observation that would otherwise have been lost with a 2D rendering. The complexity of bile fluid dynamics and biliary network architecture make it difficult to study without the appropriate tools. These advances in light microscopy technologies now allow exciting questions of the biliary network to be addressed dynamically, *in vivo* and extend this examination to human samples.

## Is Hepatic Spatial Heterogeneity Conserved From Rodent to Humans?

Most liver zonation studies were performed in rodent models (mice and rats). However, whether the findings are relevant to human hepatic physiology is a critically important question. Examination of human liver zonation was confined to light microscopy and immunohistochemistry in the 1980s (Horn et al., 1988; Hall et al., 1989). Those directly comparing human and rodent lobules observed similar heterogeneity in terms of glycogen deposits (Chamlian et al., 1989), pericentral drug-induced injury (Birge et al., 1990), and pericentral apoptotic bodies (Benedetti et al., 1988). More recently, immunofluorescence revealed that Kupffer cells were localized in periportal areas of both mice and humans, indicating the non-uniform distribution of immune cells is conserved (Gola et al., 2021). However, given the differences in metabolic demands between human and rodents, it is reasonable to speculate that some variations in metabolic compartmentalization will arise. Indeed, while certain enzymes maintain a similar lobular distribution in both human and rodents, there are multiple examples where a different pattern of expression emerges. One example is glutamine synthetase and carbamoylphosphate synthetase that participate in nitrogen metabolism, that overlap in pericentral and periportal regions, respectively, in rat liver (Gebhardt and Mecke, 1983; Moorman et al., 1989). However, the human liver contains an intermediate zone where neither enzyme is present (Multhaupt et al., 1987; Moorman et al., 1989). Similarly, the gluconeogenesis enzyme phosphoenolpyruvate carboxykinase (PEPCK) is periportally located rats (Wimmer et al., 1990b), where in humans it appears to be ubiquitous across the lobule highlighting important differences in regulation of these pathways across species (Wimmer et al., 1990a).

The apparent discrepancies between humans and rodents may also be methodological rather than physiological. Limited access to human samples introduces inherent variability that is otherwise absent in tightly controlled rodent studies; variations in sex, body composition, age, as well as samples obtained from deceased or surgical patients introduce disease/ischemia to the long list of variables. Further, older methods examining human liver heterogeneity have been limited by the throughput. Single cell and bulk RNA sequencing has allowed a vast array of genes involved in liver zonation to be examined, which is particularly pertinent for human tissues where the amount of sample obtained can often be a limiting factor in the type analyses ran. Maximizing the obtainable information from these limited samples, McEnerney et al. (2017) performed RNA-seq on human livers obtained from lean patients undergoing hepatectomy surgery, where zones of the lobule were separated via laser capture microdissection. They observed robust overlap of zone-dependent gene expression profiles between human and mice (Braeuning et al., 2006), with hypoxia and Wnt signaling pathways being key regulators of pericentral zonation. While processes such as ammonia detoxification and xenobiotic metabolism were similarly zonated for humans and rodents, aldehyde dehydrogenase (an enzyme involved in alcohol metabolism) was upregulated in periportal regions of mice but pericentral regions in humans. Expanding on this, scRNA-seq was applied to dissociated healthy human liver samples (MacParland et al., 2018), and the subsequent datasets were cross-referenced with the single cell gene expression map established in mice (Halpern et al., 2017). This provided a zonated map of both hepatocytes and non-parenchymal cells in human liver, demonstrating significant human and rodent overlap of zonation profiles. Notably, pathway analysis revealed that not all gene expression clusters had strong correlation with the established murine zonation profiles, with authors suggesting that these differences may be due to different landmark genes that define human and rodent heterogeneity. Based on scRNA-seq data, Aizarani et al. (2019) suggested there is limited evolutionary conservation of hepatic zonated gene expression between human and mice (only 68% positively correlated), despite similar zonation of well-established zonated genes such as *PCK1* (gluconeogenesis) and *CYP2A1* (drug metabolism). Similarly, single cell analysis revealed both discordant and concordant hepatic zonation profiles were found between human and rodents; genes related to drug metabolism were both pericentrally located in humans and rodents, but lipogenic genes were pericentrally located in humans and periportally located in mice (Massalha et al., 2020). However, there is discordance between functional activity and genetic expression; functional assays have demonstrated lipogenesis to be a pericentral dominant process in rats (Guzmán and Castro, 1989). This highlights potential divergent zonation gene expression profiles among species, revealing that gene expression does not always align with function, underscoring the need for more functional assessments of zonated processes, particularly in the mouse liver. The higher resolution of single cell transcriptomics may further highlight the conflicting zonation profiles between species and underlines the need for further comparisons to be made.

The paucity of human liver zonation studies is further compounded by the lack of human disease samples. The limited number of studies examining human liver zonation are typically not directly comparing NAFLD vs. non-NAFLD subjects, making it difficult to identify how liver zonation is affected by disease progression in humans. Addressing this, spatially resolved lobules were examined from liver biopsies obtained from patients undergoing oncologic or bariatric surgeries with varying degrees of severity on the NAFLD to NASH spectrum (Segovia-Miranda et al., 2019). Confirming histological observations from rodent studies, multiphoton microscopy and 3D reconstruction demonstrated that lipid accumulated in pericentral regions during early stages of NAFLD development but expanded toward periportal regions during disease progression. Additionally, disruption in nuclear texture and the bile canaliculi were observed across the lobule with NASH development. These structural alterations are indicative of dysfunction; however, further mechanistic studies are necessary to fully understand whether those are primary or secondary to NAFLD pathophysiology. An additional challenge lays in the fact that mouse models of NAFLD and NASH are unable to replicate the human disease (Takahashi et al., 2012; Van Herck et al., 2017). Therefore, identifying the differences between human and rodent metabolic compartmentalization would allow us to; (1) be aware of the caveats of extrapolating rodent results to humans, and (2) design better and more comparable rodent models to study liver zonation.

**FIGURE 3:**
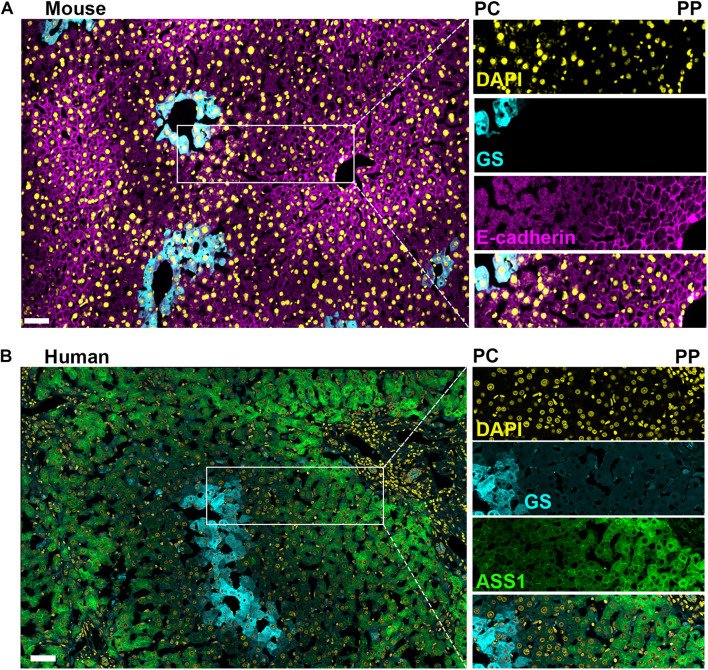
Comparison of mouse and human liver lobules. Confocal microscopy image of immunofluorescence-stained mouse and human liver sections illustrate the conservation and differences between species regarding markers of hepatocyte heterogeneity. **(A)** Mouse liver section with a pericentral (PC) to periportal (PP) axis highlighted showing; a uniform nuclei distribution across the axis (DAPI in yellow), glutamine synthetase (GS) is selectively expressed in pericentral hepatocytes (cyan), and E-cadherin is enriched in periportal hepatocytes (magenta). **(B)** Human liver section with a pericentral (PC) to periportal (PP) axis highlighted showing; a uniform nuclei distribution across the axis (DAPI in yellow) and glutamine synthetase selectively expressed in pericentral hepatocytes (cyan), while argininosuccinate synthase 1 (ASS1, green) is expressed predominantly in periportal hepatocytes. Pericentral expression of glutamine synthetase is conserved between murine and human. Scale bar = 50 μm.

Collectively, the gross similarities between rodent and human liver zonation merit the continued use of rodent models for examining the factors regulating metabolic compartmentalization in the lobule (Figure 3). However, the subtle differences described here indicate that a push for examining human liver lobules should supplement the foundational knowledge that rodent studies have provided. The coupling of mechanistic animal studies and observational human samples are important for furthering our understanding of the effects of disease progression on liver zonation and vice versa.

## Conclusion

The liver’s complex architecture gives rise to the profound heterogeneity seen among hepatocytes. This heterogeneity was astutely observed by pioneers in cell biology, whose findings have stood the test of time close to a century later. Their seminal work has created a strong foundation for hepatic physiology upon which the current body of knowledge is built.

As of late, researchers are lending more credence to this heterogeneity recognizing that investigation of the homogenized liver undermines its complexity, causing the loss of vital information. The introduction of omic approaches have vastly increased both transcriptional and translational throughput, and advancements in high resolution *in vivo* imaging has brought the study of liver heterogeneity to exciting new heights (Figure 2). Despite these advances, there are still significant gaps in our knowledge that require further investigation. While transcriptional regulation of hepatic gene expression may provide the central basis to liver zonation, additional, less explored mechanisms may be at play; these include regulation at the protein expression level and protein post-translational modifications. Although measuring protein expression and post-translational modifications requires significant larger samples, up and coming technologies such as Mass Spectrometry Imaging (MSI) address these issues and allow spatial detection of proteins, metabolites, lipids and glycans in intact tissues (Buchberger et al., 2018). Additionally, Mass Spectrometry metabolomics has recently advanced to permit analysis of single cells (Duncan et al., 2019; Thiele et al., 2019), making this the next frontier for studies of liver zonation. Notably, technologies with increased spatial and temporal resolution that do not require tissue dissociation will be superior to those that require dissociation. In this sense, light microscopy modalities and IVM would enable deep tissue 3D reconstruction and time-dependent physiological processes across the different zones of the intact liver. Importantly, microscopy permits the interrogation of the interactions between non-parenchymal cells and hepatocytes which are only starting to be resolved. Further studies are required to unravel the complex interplay between LSECs, immune cells and the microbiome in determining hepatic compartmentalization. Ultimately, advancing our understanding of metabolic compartmentalization will allow us to examine these questions in the context of liver disease that will increase both the accuracy and efficacy of future treatments.

## Author Contributions

Both authors listed have made a substantial, direct and intellectual contribution to the work, and approved it for publication.

## Conflict of Interest

The authors declare that the research was conducted in the absence of any commercial or financial relationships that could be construed as a potential conflict of interest.

## Publisher’s Note

All claims expressed in this article are solely those of the authors and do not necessarily represent those of their affiliated organizations, or those of the publisher, the editors and the reviewers. Any product that may be evaluated in this article, or claim that may be made by its manufacturer, is not guaranteed or endorsed by the publisher.
